# Decision processes underlying effort avoidance and their relationship with metacognition

**DOI:** 10.1162/IMAG.a.1332

**Published:** 2026-08-14

**Authors:** Kazuki Yoshida, Ryuji Saito, Ryota Hayashi, Seiichi Watanabe, Hiroki Okada

**Affiliations:** Faculty of Health Sciences, Hokkaido University, Sapporo, Japan; Department of Occupational Therapy, School of Rehabilitation Sciences, Health Sciences University of Hokkaido, Ishikari, Japan; Department of Occupational Therapy, Faculty of Rehabilitation, Kansai Medical University, Hirakata, Japan; Medical Corporation Nasukougen Hospital, Nasu-machi, Japan; Department of Occupational Therapy, School of Rehabilitation, Hyogo Medical University, Hyogo, Japan

**Keywords:** electroencephalography (EEG), neural decoding, representational similarity analysis, metacognition, effort-based decision making, effort avoidance

## Abstract

Effort-based decision making relies on cost–benefit computations that require individuals to evaluate expected rewards against the effort needed to obtain them. However, the temporal dynamics underlying the neural processing of reward and effort, as well as the influence of metacognitive ability on this process, remain unclear. The present study investigated these dynamics by recording electroencephalogram (EEG) data while participants performed an effort-based decision-making task and by assessing their metacognitive ability. Decoding analyses of the EEG data revealed that neural representations of effort-avoidance decisions emerged early (approximately 260 ms after stimulus onset) and remained stable over time. Crucially, representational similarity analysis showed that the brain processes reward information before effort information. Although both types of information were primarily represented in parietal electrodes, effort processing engaged a broader network that also included frontal regions. Behaviorally, higher metacognitive ability was associated with better task performance and reduced sensitivity to immediate rewards. These findings suggest a sequential decision-making process in which individuals first evaluate potential rewards and subsequently integrate information about the required effort when deciding whether to avoid effort. Furthermore, higher metacognitive ability appears to buffer against immediate reward impulses, thereby promoting long-term goal-directed behavior. Overall, this study provides direct neural evidence for the temporal dynamics of cost–benefit computation and highlights the critical role of metacognition in effort-based decision making.

## 1 Introduction

Our daily lives involve a continuous series of decisions (Baumeister et al., 1998; Mobbs et al., 2018), and the frequency of these decisions is increasing alongside the growing volume of information to which we are exposed (Lorenz-Spreen et al., 2019). The foundation of decision making is cost–benefit calculation, whereby individuals evaluate the rewards associated with a decision against the costs required to obtain them (Bogdanov & Pizzagalli, 2025; Kurzban et al., 2013; Shenhav et al., 2017; Silvestrini et al., 2023; Westbrook & Braver, 2015). In this calculation, the effort that must be expended is aversive and unpleasant and is treated as a cost, thereby decreasing the overall subjective value of the available options. Although earlier studies focused primarily on physical effort, this framework has increasingly been extended to cognitive and mental effort (Bogdanov & Pizzagalli, 2025; David et al., 2024; Shenhav et al., 2017; Westbrook & Braver, 2015).

Accumulating convergent evidence from neuroimaging studies indicates that overall subjective value is represented by a network that includes the ventral striatum (VS) and ventromedial prefrontal cortex (vmPFC) (Aridan et al., 2019; Bartra et al., 2013; Basten et al., 2010), while the roles of the anterior cingulate cortex (ACC), insula, and amygdala have been emphasized in effort-based valuation (Kurniawan et al., 2013; Lockwood et al., 2022; Müller et al., 2021). These studies have examined the comparison of reward and effort and their integration into subjective value during the decision-making process (Basten et al., 2010; Bogdanov & Pizzagalli, 2025; Brassard et al., 2024; Klein-Flügge et al., 2016; Kurniawan et al., 2013), implicitly suggesting an underlying process in which reward and effort are processed and integrated either serially or in parallel. However, several aspects of this process—particularly the temporal dynamics underlying the processing of this information—remain unclear. Clarifying these temporal dynamics would provide a deeper understanding of reward and effort processing during decision making, for example, whether these types of information are processed serially or in parallel and which process precedes the other, thereby offering critical insights into how and when decisions emerge.

Additionally, effort-based decision making extends beyond simple cost–benefit calculations; it requires assessing one’s capacity to bear these costs (in terms of physical and cognitive resources), evaluating performance based on feedback, and adapting behavior for future tasks. The ability to objectively monitor, evaluate, and regulate one’s cognition and actions is known as metacognition, which is a critical component of adaptive behavior (Fleming et al., 2012; Walker et al., 2023). Indeed, metacognition contributes to attention allocation, effective learning, and the prioritization of task performance (Aguilar-Lleyda et al., 2020; Frömer et al., 2021; Yoshida & Saito, 2025). A substantial body of research on the neural basis of metacognition has demonstrated the involvement of regions including the vmPFC, dorsomedial prefrontal cortex (dmPFC), ACC, lateral prefrontal cortex, frontal pole, and precuneus—areas that partially overlap with the neural networks underlying effort-based valuation described above (Bang & Fleming, 2018; Fleur et al., 2021; Qiu et al., 2018; Seow et al., 2025).

Substantial individual differences have been reported in metacognitive accuracy and confidence ratings (Rahnev, 2025; Rouault, McWilliams, et al., 2018), which may contribute to variation in decision-making processes and choice behavior. Evidence suggests that individuals with lower confidence in their own abilities (e.g., memory) are more likely to engage in cognitive offloading—that is, relying on external devices to reduce cognitive load (Gilbert et al., 2020; Hu et al., 2019). Considering that both cost–benefit calculation and metacognitive abilities are often impaired in neurological and psychiatric disorders (Saleh et al., 2023; Yoshida et al., 2023), understanding how these functions interact to shape behavior is crucial. Nonetheless, studies investigating the relationship between metacognition and effort-based decision making, including effort avoidance, remain limited, leaving the precise nature of these associations unclear. Therefore, uncovering both the temporal dynamics of cost–benefit integration and their metacognitive correlates is critical for establishing a unified framework of adaptive effort allocation and avoidance behaviors.

To achieve a unified understanding of effort avoidance and the role of metacognition, the present study aimed to elucidate the temporal dynamics of reward and difficulty processing during effort-based decision making. Beyond examining temporal processing, we further investigated how individual differences—specifically metacognitive ability—are associated with task performance and the frequency of effort avoidance. To address these interconnected aims, we measured participants’ metacognitive abilities using a behavioral task and recorded electroencephalogram (EEG) data while they performed an effort-based decision-making task. Using the EEG data, we conducted decoding analyses based on machine learning techniques to examine the temporal dynamics of effort-avoidance decisions. Furthermore, we employed representational similarity analysis (RSA), a multivariate approach that assesses whether neural activity patterns reflect specific model structures (e.g., reward and difficulty), to determine the precise time windows during which reward and difficulty information are processed. We hypothesized that reward and difficulty information would exhibit distinct temporal dynamics and that higher metacognitive ability would be correlated with better performance on effort-based decision-making tasks.

## 2 Methods

### 2.1 Participants

The required sample size was calculated using G*power 3.1. We determined a target sample size of 34 participants to detect a moderate effect size (ρ = 0.4) with a significance level (α) of 0.05 and a statistical power of 0.80.

In total, 36 right-handed healthy young participants were recruited for this study. All participants had normal or corrected-to-normal vision and no history of neurological or psychiatric disorders. The study protocol was approved by the ethics committee of the Faculty of Health Sciences at Hokkaido University (approval number: 23–93) and was conducted in accordance with the Declaration of Helsinki. All participants provided written informed consent.

### 2.2 Experimental procedure

**Fig. 1.**
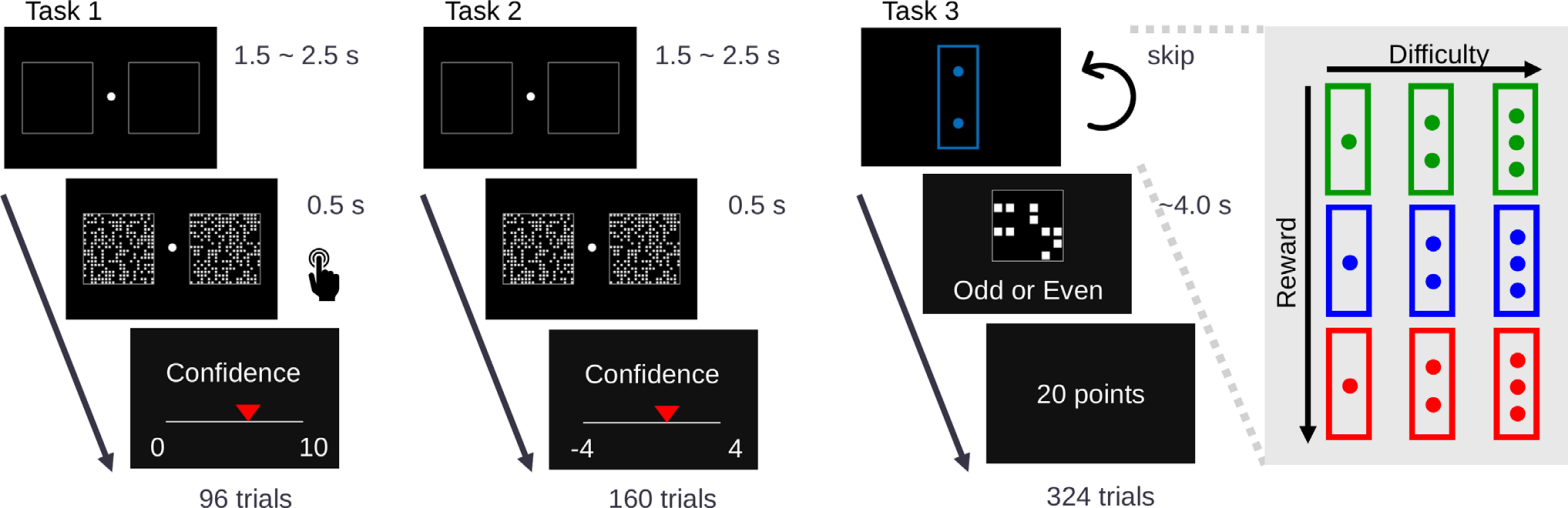
Outline of the experimental tasks. In Task 1, the difficulty level corresponding to approximately 75% accuracy in the perceptual discrimination task was determined. In Task 2, this difficulty level was fixed. Participants indicated both their perceptual discrimination judgments and confidence ratings simultaneously by assigning positive or negative values to their responses. Task 3 involved a cognitive effort-avoidance task in which participants decided whether to engage in an odd/even judgment task after reviewing information regarding rewards (points) and difficulty.

A behavioral task was created and presented using PsychoPy (v2024.1.5) (Peirce et al., 2019). Participants were seated 60 cm away from a 23-inch monitor with a resolution of 1920 × 1080 pixels. An outline of the experimental tasks is shown in Figure 1. The experiment comprised three tasks with distinct roles: Task 1 was an adaptive staircase procedure used to calibrate each participant’s perceptual discrimination threshold; Task 2 presented this individually calibrated, fixed-difficulty discrimination task with trial-by-trial confidence ratings, from which the metacognitive indices (d′, meta-d′, and m-ratio) were computed; and Task 3 was the cognitive effort-avoidance task during which EEG was recorded.

In Task 1, participants were first presented with two squares containing differing number of white dots for 0.5 s. Participants judged which of the two squares contained a larger number of dots by pressing 9 (left) or 0 (right) as quickly as possible. One of the two squares contained 200 dots, whereas the other square contained a larger number. Task difficulty was defined as the difference in the number of dots between the left and right squares (2, 4, 8, 16, 24, 32, 48, and 64 dots; smaller differences indicated greater difficultly) and was controlled using the 1-up 2-down staircase procedure. The staircase began at a difference of 16 dots (the fourth most difficult level). After two consecutive correct responses, the difference was decreased by one step (increasing difficulty), whereas after a single incorrect response, it was increased by one step (decreasing difficulty).

After each perceptual discrimination judgment, participants reported their confidence in their judgment using a visual analog scale ranging from 0 (low confidence) to 10 (high confidence). Participants completed 2 blocks of 48 trials each, for a total of 96 trials. In this task, the difficulty level that yielded an accuracy rate of approximately 75% was used in Task 2.

Task 2 was almost identical to Task 1, but participants reported their confidence judgments on a scale ranging from -4 to 4 (excluding 0), thereby simultaneously indicating their perceptual discrimination judgment and the magnitude of their confidence rating. Negative and positive values indicated selection of the left and right stimuli, respectively. The absolute value represented the level of confidence (e.g., selecting the left stimulus with the highest confidence corresponded to a rating of -4). Task 2 consisted of 4 blocks of 40 trials each, for a total of 160 trials.

Although EEG data were recorded during this task, they are outside the scope of the current study and are, therefore, not reported. Because estimation of metacognitive efficiency (m-ratio) assumes approximately constant task difficulty (Maniscalco & Lau, 2012; Rahnev & Fleming, 2019), the metacognitive indices were derived exclusively from this fixed-difficulty task (Task 2). The adaptive staircase procedure used in Task 1 served only to calibrate difficulty and was, therefore, not used to estimate metacognition.

Subsequently, participants performed Task 3, the cognitive effort-avoidance task, while EEG was recorded. Task 3 was developed based on previous studies (Khalighinejad et al., 2021; Lockwood et al., 2024; Müller et al., 2022; Saleh et al., 2023), but replaced the physical-effort task used in those studies with a counting task requiring cognitive effort.

In Task 3, participants were presented with a diagram indicating the rewards (points) and difficulty associated with the upcoming counting trial. Rewards were signaled by the color of the shape (green, 5 points; blue, 10 points; red, 20 points), whereas difficulty was indicated symbolically by the number of dots displayed within the shape (1, 2, or 3 dots indicating low, medium, and high difficulty, respectively). These preview dots served as symbolic difficulty cues and were not the dots to be counted in the subsequent task.

Participants could visually assess the reward and difficulty associated with the upcoming trial and decide whether to accept or avoid it based on their evaluation. To accept the task, participants pressed the 9 key, whereas pressing the 0 key allowed them to avoid it. The subsequent task required participants to count white dots randomly arranged within a 6 × 6 grid and indicate whether the total number of dots was even (0 key) or odd (9 key). The number of dots was determined by the difficulty level indicated in the cue (low, 1–4 dots; medium, 7–10 dots; high, 13–16 dots).

If the response was correct, participants earned the number of points indicated by the cue color. These points constituted the only reward, and participants were instructed to maximize their total score. There was no time limit for the accept/avoid decision. After the decision, a jittered interval of 1–2 s preceded the onset of the counting task, during which participants reported whether the number of dots was odd or even within 4 s. Feedback was subsequently presented for 1.5 s.

There were nine stimulus combinations consisting of three reward conditions and three difficulty conditions. Participants completed 6 blocks of 54 trials each, resulting in a total of 324 trials. All stimuli were presented in a random order.

At the end of the experiment, participants rated the subjective value of engaging in each condition (the 9 reward–difficulty combinations) using a visual analog scale ranging from 0 to 10.

### 2.3 EEG recording and preprocessing

EEG data were recorded using an ActiCHamp Plus system (Brain Products GmbH, Munich, Germany) with active electrodes mounted on an elastic cap according to the international 10–20 system. The reference electrode was placed at the tip of the nose, while the ground electrode was positioned at AFz. A total of 63 electrodes were positioned at the following sites: Fp1, Fp2, AFz, AF3, AF4, AF7, AF8, F1, F2, F3, F4, F5, F6, F7, F8, Fz, FC1, FC2, FC3, FC4, FC5, FC6, FCz, FT7, FT8, C1, C2, C3, C4, C5, C6, Cz, T7, T8, CP1, CP2, CP3, CP4, CP5, CP6, TP7, TP8, TP9, TP10, P1, P2, P3, P4, P5, P6, P7, P8, Pz, PO3, PO4, PO7, PO8, POz, O1, O2, Oz, and Iz.

Electrooculograms (EOGs) were recorded using bipolar electrodes placed at the infraorbital and supraorbital regions of the left eye, as well as at the outer canthi of both eyes. Electrode impedance was maintained below 5 kΩ, and the sampling rate was set at 500 Hz.

Brainstorm software (Tadel et al., 2019) was utilized for preprocessing and analysis of the EEG data. EEG signals were band-pass filtered between 1 and 40 Hz, re-referenced to the average reference, and subjected to independent component analysis to remove ocular artifacts. EEG epochs were time locked to the onset of the reward-and-difficulty cue (i.e., the offer diagram; t = 0) and spanned from −200 to 1200 ms.

### 2.4 Behavioral analysis

First, we calculated metacognitive efficiency (m-ratio), and metacognitive bias (mean confidence ratings) from the Task 2 data. To quantify participants’ metacognitive efficiency, we computed discriminability (d’) using signal detection theory and metacognitive sensitivity (meta-d’) for each participant. Meta-d’ measures the extent to which confidence ratings discriminate between correct and incorrect judgments. Subsequently, we calculated the m-ratio (meta-d’/ d’). We modified and used the MATLAB code available at http://github.com/smfleming/HMM (Fleming, 2017) to calculate these parameters from the rating data. Although this toolbox implements a hierarchical model, meta-d′ was estimated individually for each participant rather than within the group hierarchy so that the resulting m-ratios remained independent for use in subsequent analyses.

Next, from the cognitive effort-avoidance task, we extracted three measures: the total score obtained in the task (log-transformed), the task acceptance rate, and the reaction time during the accept/avoid decision. Furthermore, to understand the extent to which each participant was influenced by reward and difficulty information during decision making, we calculated reward sensitivity and effort sensitivity using the following equations:

SV=k+αReward+βEffort+γReward:Effort (1)

$$p=1/1+e^{-SV},$$ (2)

where SV represents the subjective value and p  denotes the probability of accepting the task offer. The parameter *k* reflects the general tendency to accept an offer. α indicates reward sensitivity, representing responsiveness to rewards, whereas β represents effort sensitivity, reflecting aversion to increased effort. γ serves as the interaction term between reward and effort. Typically, α takes a positive value, meaning that SV increases as rewards increase, whereas β takes a negative value, indicating that SV decreases as effort increases. The model posits that the probability of acceptance increases as SV increases. We fitted this model to each participant’s choice data using maximum likelihood estimation by minimizing the negative log-likelihood of the observed accept/avoid choices with the Nelder–Mead simplex algorithm (fminsearch, MATLAB).

### 2.5 Decoding analysis and temporal generalization analysis

Multivariate time-series decoding was performed to classify acceptance versus avoidance trials using a linear support vector machine (SVM), implemented using the CoSMoMVPA toolbox (Oosterhof et al., 2016) and LIBSVM (Chang & Lin, 2003) in MATLAB 2019b (MathWorks, MA, USA). Whole-brain decoding analysis included all EEG electrodes (i.e., 63 channels) using a sliding time window of 20 ms. The data were partitioned into four folds, and data balancing was performed within the training set to ensure an equal number of trials for both conditions.

To improve the signal-to-noise ratio (SNR) of the training data, pseudo-trials were generated by averaging five randomly selected trials from each condition. This resampling procedure was repeated 20 times to create a robust training set. The trained classifier was then applied to the independent test fold to calculate classification accuracy. This procedure was repeated four times (fourfold cross-validation) to obtain time-resolved decoding accuracies for each participant.

To investigate whether the neural representations underlying the acceptance and avoidance of offers remained stable or changed over time, we conducted a temporal generalization analysis using multivariate pattern analysis (MVPA). In this approach, a classifier is trained on data from a specific time point and tested on data from all other time points (i.e., generalization times). If neural representations remain stable over time, the classifier should maintain high-classification accuracy across different time points. Conversely, if classification accuracy does not generalize to other time intervals, this suggests that the representational format has changed. This analysis was performed for each participant, and the resulting classification accuracies were averaged across participants.

### 2.6 RSA and searchlight analysis

RSA was employed to examine the neural representations of reward and effort information, which were considered relevant to acceptance and avoidance decisions. First, we computed model representational dissimilarity matrices (RDMs) of size 9 × 9 for reward information, effort information, and their integrated information (defined as the Euclidean distance between stimuli). These models were derived from the reward and difficulty levels associated with each stimulus.

The neural RDM was constructed based on the pairwise decoding accuracy between stimuli calculated from the EEG data. In this context, higher decoding accuracy indicates that two stimuli are more clearly distinguishable, corresponding to greater representational dissimilarity. To reduce computational demands, we employed a sliding analysis window of 52 ms with a step size of 20 ms. Classification was performed using linear discriminant analysis (LDA) on the full training dataset without trial averaging. LDA was selected because it is computationally efficient, robust to noise, and well suited to RSA frameworks (Blankertz et al., 2011; Grootswagers et al., 2017; Walther et al., 2016).

To investigate the spatial distribution underlying the RSA results—specifically to identify which channels were involved in processing reward and effort information—we conducted a time-resolved channel-space searchlight analysis. For each EEG channel, we defined a local cluster consisting of the target channel and its four nearest neighbors and performed decoding analysis using these channels. The resulting representational similarity (i.e., the correlation coefficient between the neural RDM and the model RDM) was assigned to the center channel of the cluster. We specifically focused on time windows during which only reward information, both types of information, or only effort information was represented, and generated searchlight maps for each of these periods.

Finally, as an exploratory analysis, we investigated whether the processing of reward and effort information differed between participants with high- and low-metacognitive efficiency (m-ratio). Participants were divided into high- and low-metacognitive-efficiency groups using a median split, and RSA was conducted separately for each group. The RSA procedures were identical to those described above.

### 2.7 Independence from visual, reaction-time, and motor confounds

To test whether reward- and effort-related neural responses arise independently of low-level visual features, reaction-time, and motor (key-press) responses, we performed four additional control analyses. Neural dissimilarity between conditions was quantified using the cross-validated Mahalanobis distance (crossnobis; Walther et al., 2016), a cross-validation–based measure that is consistent with the cross-validated LDA decoding used in the main RSA. The time windows and sliding-window procedure were identical to those used in the main RSA. A neural RDM was computed for each time window and participant, and these same neural RDMs were used in the three RSA-based control analyses described below.

The first control analysis tested the subject specificity of subjective-value representations. Because stimulus features such as color and numerosity were identical across participants, any stimulus-driven representation can contribute only a participant-common component, whereas subjective value differs across participants. We exploited this asymmetry to test whether reward and effort processing was subject specific. For the 27 participants with available subjective-value ratings, the subjective-value model RDM was defined as the absolute difference in ratings between conditions (|SV_a − SV_b|). For each participant and time window, we computed the Fisher z-transformed Spearman correlation between the participant’s neural RDM and their own model RDM (z_own), together with the mean Fisher z-transformed Spearman correlation between their neural RDM and every other participant’s model RDM (z_other), and calculated the difference, Δ = z_own − z_other. A positive Δ indicates that the neural representation reflects each participant’s idiosyncratic subjective value—which cannot, in principle, arise from visual features that were identical for all participants—thereby identifying the time windows during which neural representations cannot be explained by stimulus-driven visual processing.

The second control analysis dissociated reward representations from color representations. In our task, reward magnitude was signaled by cue color. We, therefore, converted the cue colors into the perceptually uniform CIELAB space and computed the distances between them to construct a color model RDM. The perceptual distance structure of the colors differed from the distance structure of reward magnitude, so the two models predicted distinct neural geometries. For example, the low- and medium-reward cues, which were closest in reward magnitude, were the most distant in color space. For each participant and time window, we computed Spearman correlations between the neural RDM and each model RDM (reward and color) and directly compared the fits of the two models. Because this analysis did not require subjective-value ratings, it included all participants in the EEG analysis (n = 30). This analysis allowed us to assess whether the reward representation was confounded by color, a low-level visual feature.

A third RSA-based control analysis tested whether the reward and effort representations were confounded by reaction time (RT), which differed across conditions and covaried with difficulty. For each participant, we constructed an RT model RDM as the absolute difference in mean RT between conditions (|RT_a − RT_b|) and recomputed the reward and difficulty RSA using partial Spearman correlations that controlled for this RT model RDM (n = 30). If a model continued to predict the neural RDM after partialling out RT, the representation could not be attributed to between-condition RT differences.

Finally, we performed a cross-decoding analysis (n = 30) to control for motor confounds; that is, to test whether the neural signal distinguishing accept from avoid decisions was contaminated by motor preparation associated with the two response keys. Using key-press data from the counting task (epoched from −400 to 200 ms relative to the key press), we trained a classifier to discriminate between the two keys (0 vs. 9). Because the keys differed in trial number, training trials were subsampled to match the smaller class, and this procedure was repeated five times and averaged. The accept/avoid test data were epoched identically and left unbalanced.

In a response-locked analysis, a regularized LDA trained on the counting-task key-press data at each time point was tested on the accept/avoid data at the corresponding time point. In a cue-locked analysis, a regularized LDA trained on counting-task data averaged over ± 100 ms around the key press, was applied to each time point of the cue-locked accept/avoid epoch. Because the training and test data originated from different tasks—and, in the cue-locked analysis, from different temporal reference points—decoding performance was quantified using the area under the receiver operating characteristic curve (AUC), which is independent of the classification threshold and relatively insensitive to class imbalance. Together, these analyses identify the time windows during which key-press–related motor activity contaminates the decision signal.

### 2.8 Statistical analysis

To validate the behavioral task, we examined whether metacognitive ratings differed significantly between correct and incorrect trials in the perceptual discrimination task. We also tested whether acceptance rates increased with higher rewards and decreased with greater effort (difficulty). These analyses were performed using paired t-tests and repeated measures ANOVAs, respectively.

Furthermore, we calculated Pearson’s correlation coefficients between metacognitive efficiency (m-ratio), metacognitive bias, effort-avoidance task scores, acceptance rates, and the model parameters (k,α,β ). Finally, one-sample t-tests were conducted to determine whether reward and effort sensitivities differed significantly from zero at the group level.

To identify significant time windows for classification accuracy and correlation coefficients in the decoding, RSA, and control analyses (Section 2.7), we employed threshold-free cluster enhancement (TFCE) (Mensen & Khatami, 2013), correcting for multiple comparisons across time (E = 0.5, H = 2, dh = 0.1). For the main decoding and RSA, null distributions were generated by randomly permuting condition labels (2,000 iterations), as implemented in the CoSMoMVPA toolbox.

For the four control analyses (Section 2.7)—that is, group-level tests of participant-level statistics against chance—null distributions were instead generated by sign flipping each participant’s statistic across participants (Δ = z_own − z_other for the own-versus-other analysis; the Fisher z-transformed Spearman correlation for the reward–color analysis; the partial Spearman correlation for the RT analysis; and AUC − 0.5 for the motor cross-decoding) and recomputing the maximal TFCE score on the resulting one-sample t-map, repeated 2,000 times. Tests were one sided (effect > chance), except for the color model analysis, which was two sided.

In the reward-versus-color analysis, we additionally compared the two models directly. For each participant, the reward- and color-model fits were averaged within the a priori early time window, and the difference (reward − color) was tested against zero using a paired t-test. All statistical analyses were performed using R (version 4.3.0) or MATLAB (2019b), with the significance level set at 0.05.

## 3 Results

Participants were excluded from the analyses if their metacognitive efficiency (m-ratio) was negative (n = 1) or if their subjective value ratings (measured using a VAS at the end of the experiment) showed an inverted relationship with reward or difficulty levels (n = 2), as these patterns strongly suggested a failure to understand the task instructions. This resulted in a final sample of 33 participants (mean age = 21.88 ± 1.51 years, education = 16.3 ± 1.53, female = 15). Additionally, three participants who had no avoidance trials in the effort-avoidance task were excluded from the MVPA and RSA, leaving a total of 30 participants for these analyses (mean age = 21.96 ± 1.53, education = 16.4 ± 1.56, female = 15).

### 3.1 Behavioral results

The number of valid trials in Task 2 used to calculate the metacognitive indices averaged 158.2 ± 2.1 (maximum = 160 trials), ranging from 149 to 160 trials. The descriptive statistics (mean ± SD) for the metacognitive indices and the effort-avoidance task were as follows: m-ratio = 0.55 ± 0.33, metacognitive bias = 2.09 ± 0.48, overall acceptance rate = 79.0 ± 14.1%, and overall reaction time = 0.95 ± 0.26 s.

**Fig. 2.**
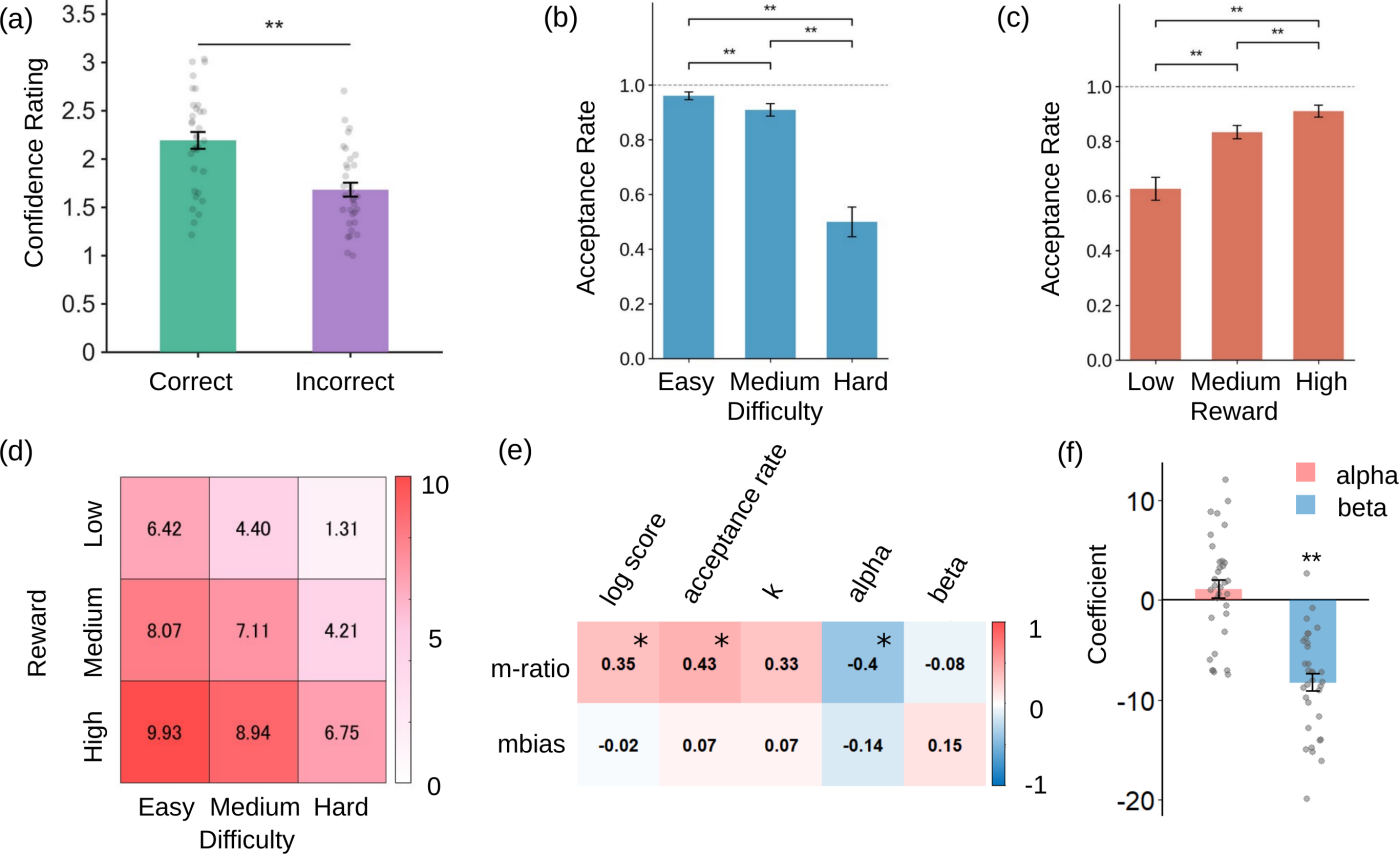
Behavioral results. Confidence ratings for correct and incorrect trials in the perceptual discrimination task (Task 2) (a). Acceptance rates across different difficulty levels in the cognitive effort-avoidance task (b). Acceptance rates across different reward levels in the cognitive effort-avoidance task (c). Bars show group means, and error bars denote ±1 SEM. Acceptance rate is bounded between 0 and 1 (dashed line at 1.0). Heatmap of the subjective value of task engagement, rated on a visual analog scale (VAS) ranging from 0 to 10 (d). Correlations between metacognition-related indices and cognitive effort-avoidance indices (e). Results of one-sample t-tests for the reward sensitivity (α) and effort sensitivity (β) coefficients (f). *p < 0.05, **p < 0.01.

Confidence ratings were significantly higher for correct discrimination trials than for incorrect trials in the metacognitive task (t(32) = 9.74, *p* < 0.001, 95% CI [0.40 0.62], d = 0.259) (Fig. 2a). We also examined how acceptance rates changed as a function of reward and difficulty, confirming that acceptance rates decrease as rewards declined (F[1.19,37.9] = 38.2, *p* < 0.001, $\eta_{p}^{2}=0.58$ ; high: mean = 0.91, SD = 0.13 > medium: mean = 0.83, SD = 0.14 > low: mean = 0.62, SD = 0.24) and as difficulty increased (F[1.15,37] = 64.8, *p* < 0.001, $\eta_{p}^{2}=0.686$ ; easy: mean = 0.96, SD = 0.08 > medium: mean = 0.91, SD = 0.13 > hard: mean = 0.50, SD = 0.32) (Fig. 2b, c).

Furthermore, the estimated reward sensitivity did not differ significantly from zero (t(32) = 1.18, 95% CI [-0.79 2.99], *p* = 0.246), whereas effort sensitivity was significantly lower than zero (t(32) = -9.43, 95% CI [-10.06 -6.49], *p* < 0.001) (Fig. 2f).

Correlation analyses revealed that metacognitive efficiency (m-ratio) showed significant positive correlations with log-transformed scores (r = 0.354, *p* = 0.043) and acceptance rates (r = 0.431, *p* = 0.012), as well as a significant negative correlation with reward sensitivity (r = -0.4, *p* = 0.022). By contrast, no significant correlations were observed between metacognitive bias and the effort-avoidance task variables (Fig. 2e).

Acceptance rates remained relatively stable across blocks (80.6%, 80.2%, 79.0%, 78.1%, 77.2%, and 79.1% for Blocks 1–6), with no significant main effect of block in a repeated-measures ANOVA (F(5,160) = 0.41, *p* = 0.84) and no significant difference between the first and second halves of the task in a paired t-test (79.9% vs. 78.1%; t(32) = 0.86, *p* = 0.40). We found no evidence of fatigue, time-on-task effects, or an early-finish strategy. Instead, choices tracked the offered reward and difficulty levels (Fig. 2b, c).

### 3.2 Time-resolved multivariate decision decoding

**Fig. 3.**
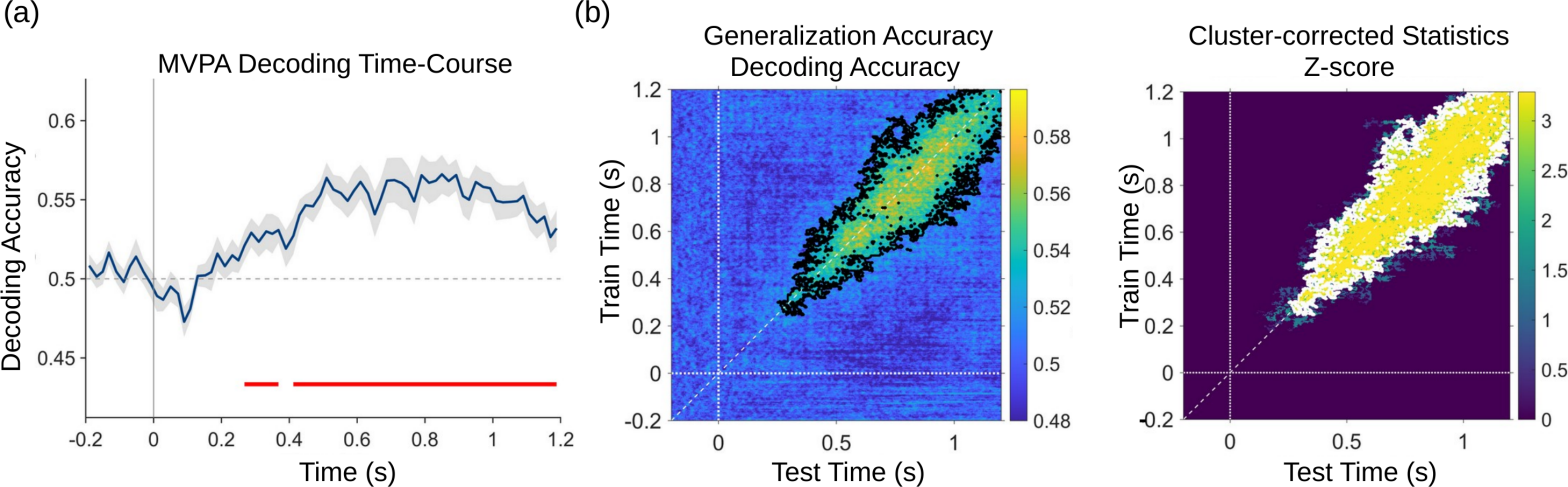
Decoding analysis results. Time-resolved decoding analysis of acceptance versus avoidance decisions. Statistically significant time windows are indicated by red lines (a). Results of the temporal generalization analysis (b). The left panel shows decoding accuracy, whereas the right panel displays cluster-corrected statistical values (z-scores). Significant regions are outlined in black or white.

Time-resolved decoding analysis of acceptance versus avoidance decisions revealed that classification accuracy reached significance at 260 ms (*p* < 0.05). Although classification accuracy decreased around 400 ms, it subsequently increased until approximately 1 s, corresponding to the period preceding the response (key press) (Fig. 3a). This finding provides crucial information regarding the timing of the neural processes underlying the decision to accept or avoid an offer and serves as an important foundation for interpreting the subsequent RSA results. The temporal generalization analysis indicated that neural processing between 250 and 400 ms was time specific and transient, whereas relatively stable neural representations emerged thereafter (Fig. 3b).

### 3.3 Representation of reward and effort revealed by RSA

**Fig. 4.**
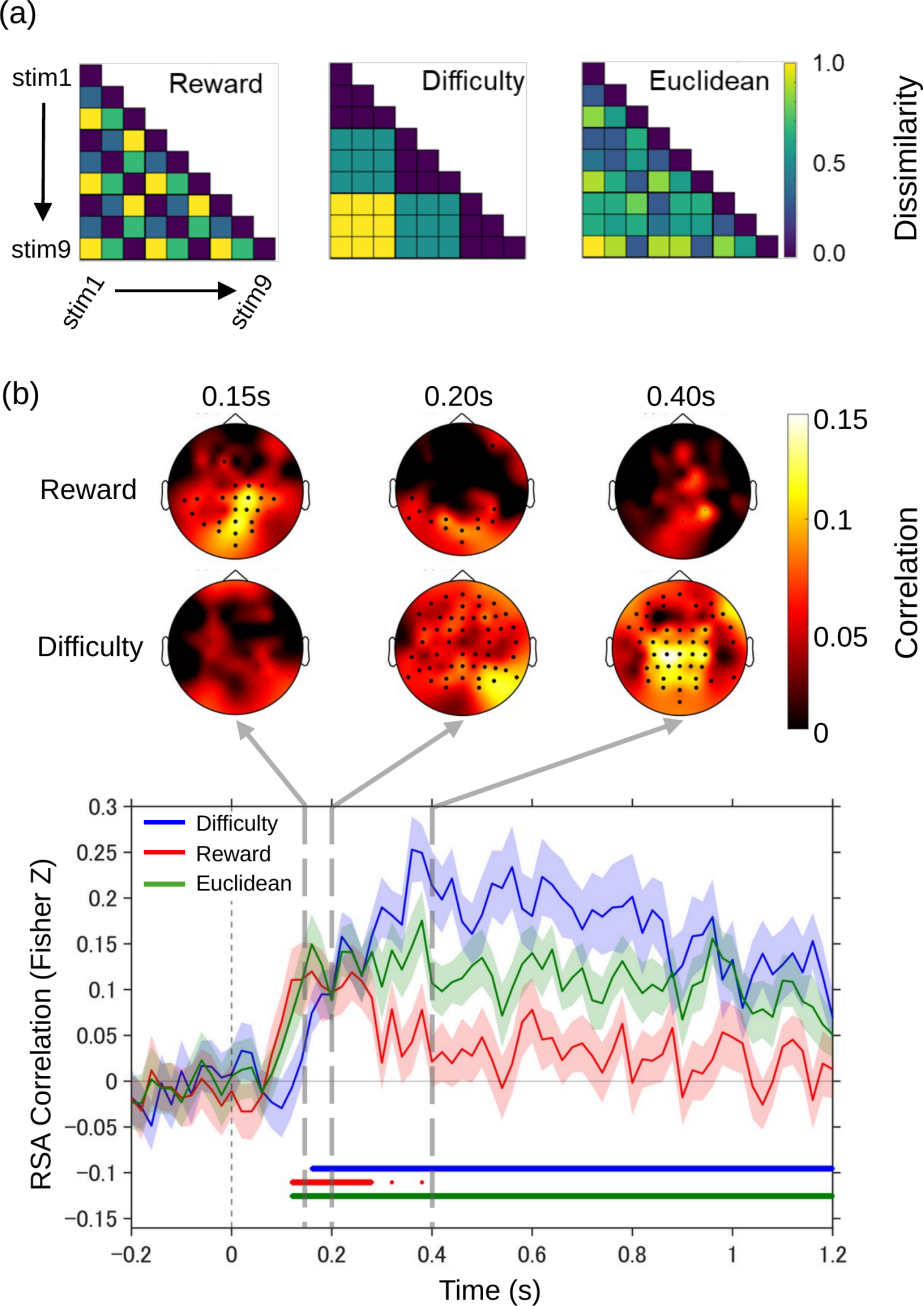
RSA results. This figure presents the model RDMs (a) and the RSA results (b). The time-series plots illustrate the time points at which the neural RDMs derived from EEG show high correlations with effort information (blue), reward information (red), and their integrated information (Euclidean distance; green) (Fisher z; mean ± SEM). Solid colored lines indicate the time intervals during which these correlations are statistically significant. The topomaps above display the results of the searchlight analysis at each time point (0.15 s, 0.2 s, and 0.4 s), with significant electrodes marked by black dots. RDM = representational dissimilarity matrix; RSA = representational similarity analysis.

Figure 4 presents the model RDMs (Fig. 4a) and the RSA results (Fig. 4b). TFCE analysis revealed that representations of reward became significant at 120 ms, whereas representations of difficulty became significant at 160 ms. While reward representations ceased to be significant around 400 ms, difficulty representations persisted until approximately 1 s, coinciding with the timing of the decision response. A model based on the Euclidean distance in the combined reward–difficulty space remained significant throughout the interval spanning the reward and difficulty representations, consistent with a representation that jointly reflects both dimensions.

Similarly, the topomaps illustrate the results of the searchlight analysis. These results indicate that reward information is represented primarily by electrodes in the parieto-occipital regions. By contrast, difficulty information is represented by a more widespread set of electrodes that includes frontal areas while remaining centered on the parietal region.

**Fig. 5.**
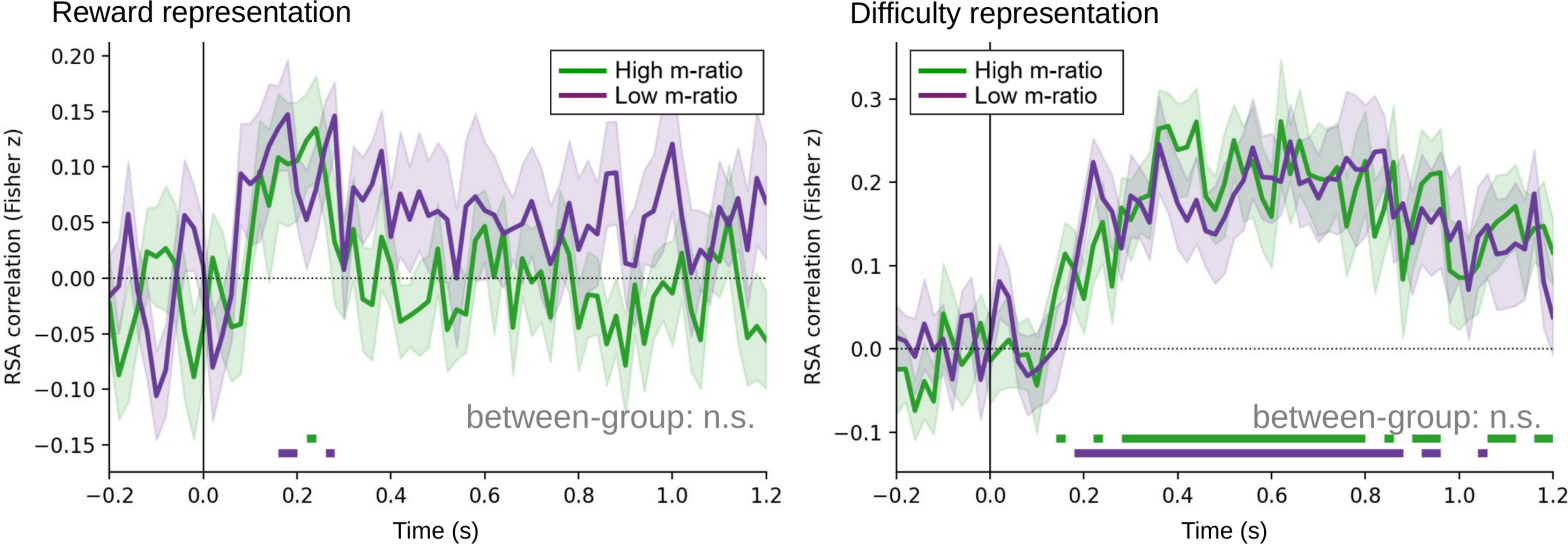
Results of the exploratory RSA for high- and low-metacognitive-efficiency (m-ratio) groups. Time-resolved RSA (Fisher z; mean ± SEM) between the whole-scalp neural RDM and the reward (left) and difficulty (right) model RDMs for the high- (green, n = 15) and low- (purple, n = 15) metacognitive-efficiency groups (median split of the same 30 participants used in the main analyses). Colored bars indicate within-group time windows of significant model–brain correlations (*p* < 0.05; green = high, purple = low). In these separate within-group analyses, reward representations reached significance earlier in the low-efficiency group (~160 ms) than in the high-efficiency group (~220 ms); however, the direct between-group comparison was not significant.

Given the observed correlation between metacognitive efficiency (m-ratio) and reward sensitivity, we conducted an exploratory analysis. Participants were divided into high- and low-metacognitive-efficiency groups using a median split (high: n = 15, low: n = 15), and the time-resolved representations of reward and difficulty information were compared between groups. In the separate within-group analyses, reward representations reached significance earlier in the low-efficiency group (160 ms) than in the high-efficiency group (220 ms; Fig. 5). However, a direct between-group comparison using TFCE permutation testing revealed no significant differences at any time point (minimum *p* = 0.48).

### 3.4 Reward and effort representations are not explained by visual, reaction-time, or motor confounds

**Fig. 6.**
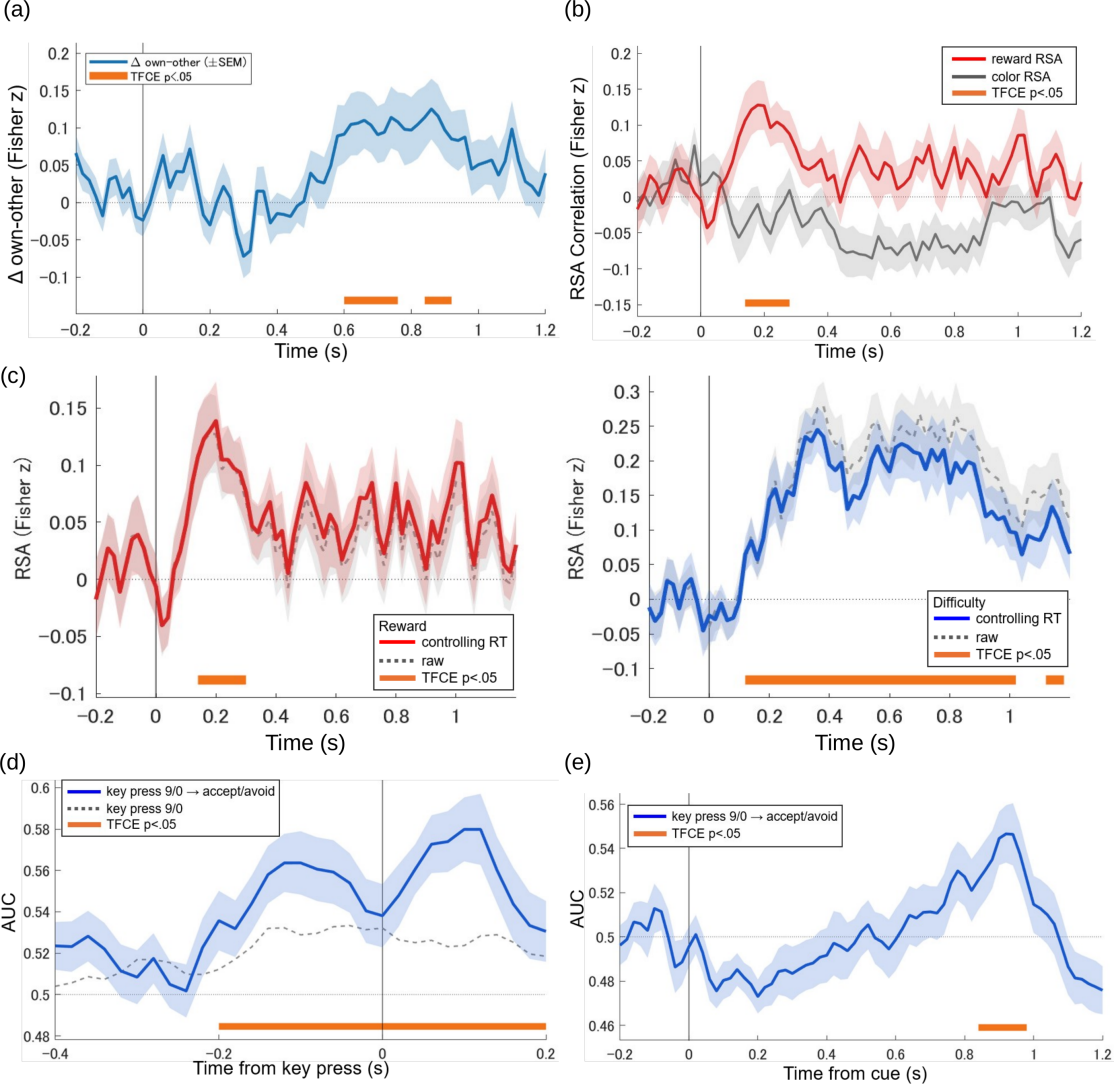
Results of the control analyses for visual, reaction-time, and motor confounds. Own-versus-other analysis: difference between the correlation of each participant’s neural RDM with their own versus other participants’ subjective-value RDMs, Δ = z(own) − z(other) (Fisher z; mean ± SEM, n = 27) (a). Reward-versus-color RSA: Spearman correlation (Fisher z) between the neural RDM and the reward RDM (red) and the color RDM (gray) (mean ± SEM, n = 30) (b). Reward (left, red) and difficulty (right, blue) representations remained significant after reaction time was partialled out; gray dashed lines show the corresponding correlations without reaction-time control (mean ± SEM, n = 30) (c). Motor cross decoding, response locked: a classifier trained on the counting-task key press (key 9 vs. 0) and applied to the decision key press (accept = 9 vs. avoid = 0); transfer performance is shown in blue (AUC; mean ± SEM, n = 30). The gray dashed line indicates within-counting-task decoding of key 9 versus key 0. The x-axis represents time relative to the key press (d). The same cross-decoding analysis is shown for the cue-locked data (blue; AUC, n = 30) (e). Orange lines denote time windows showing significant effects at TFCE *p* < 0.05.

We performed four control analyses to confirm that the main findings were not driven by low-level visual features, reaction time, or motor confounds. In the first analysis (Fig. 6a), each participant’s neural RDM was more significantly associated with their own subjective-value model RDM than with those of other participants (z_own > z_other; Δ > 0)—that is, more significantly than with the participant-common component arising from the physically identical cues—during two time windows (600–760 ms, *p* = 0.018, and 840–920 ms, *p* = 0.017, n = 27). This finding indicates that subject-specific subjective-value representations emerged relatively late, beginning at approximately 600 ms.

In the second analysis (Fig. 6b), we examined the extent to which the neural RDM correlated with the reward and color model RDMs over time. Consistent with the main analysis, the reward model RDM showed a significant positive correlation from 140 to 280 ms after cue onset, whereas the color model RDM showed no time window of significant positive correlation. A direct comparison within the early time window (140–260 ms) confirmed that the reward model provided a significantly better fit to the data than the color model (reward − color: Δz = 0.14, t(29) = 5.4, *p* < 0.001). Thus, the early reward-related representation reflects reward magnitude and cannot be explained by color perception.

In a third RSA-based control analysis (Fig. 6c), both the reward and difficulty model RDMs continued to predict the neural RDM after partialling out a reaction-time model RDM (reward, 140–300 ms, *p* = 0.0015; difficulty, 120–1020 ms, *p* < 0.001, and 1140–1160 ms, *p* = 0.029), indicating that these representations cannot be explained by reaction-time differences between conditions.

The fourth analysis examined whether the decision-related neural signal—the pattern distinguishing acceptance from avoidance—was contaminated by motor activity. In the response-locked analysis, the motor classifier transferred significantly around the key press (−200 to 200 ms, *p* < 0.001; Fig. 6d). When the same classifier was applied to the cue-locked epoch, transfer was significant only around the mean reaction time (840–980 ms after cue onset, *p* = 0.038; Fig. 6e) and remained at chance at earlier time points. These results indicate that the late increase in accept-versus-avoid decoding partly reflects shared motor activity, whereas the earlier decision representation is independent of motor-related processes.

## 4 Discussion

This study aimed to elucidate the temporal dynamics of neural representations during effort-based decision making and to investigate whether metacognitive ability is associated with these decisions and task performance. Decoding analysis of the EEG data revealed that the neural representation associated with the decision to avoid effort emerged early (from 260 ms) and that prediction accuracy increased up to the time of the actual response (~1.0 s). Furthermore, RSA demonstrated that reward information was represented earlier than difficulty information (i.e., the required amount of effort), with difficulty information being represented subsequently. Additionally, individual differences in participants’ metacognitive abilities were associated with their performance on the effort-avoidance task, supporting our hypothesis.

We examined the behavioral data to assess the validity of our metacognitive measurement task and cognitive effort-avoidance task (Fig. 2). In the metacognitive task, confidence ratings were significantly higher for correct trials than for incorrect trials in the perceptual discrimination task, and the m-ratio generally fell between 0 and 1 (Rahnev, 2025; Rahnev & Fleming, 2019). In the cognitive effort-avoidance task, the acceptance rate decreased as task difficulty increased, whereas it increased as reward increased. Although reward sensitivity showed a positive value, it did not reach statistical significance; conversely, effort sensitivity was significantly lower than zero. Furthermore, the post-task VAS ratings demonstrated that subjective value—integrating both reward and difficulty information—was accurately evaluated by the participants. Collectively, these results indicate that our metacognitive measurement and cognitive effort-avoidance tasks functioned as intended.

Importantly, metacognitive efficiency (m-ratio) was positively correlated with both the effort-avoidance task score (log-transformed score) and acceptance rate, whereas it was negatively correlated with reward sensitivity. This finding aligns with our hypothesis, suggesting that higher metacognitive ability is associated with better performance in effort-based decision-making tasks. Furthermore, the finding that m-ratio was negatively correlated with reward sensitivity—rather than effort sensitivity—suggests an alternative explanation for this behavior. Specifically, rather than reflecting an adaptive process in which individuals monitor their ability against task difficulty to choose effort-avoidant trials, this relationship may instead reflect reduced sensitivity to the offered reward magnitude.

We note that our task did not contrast immediate and delayed rewards and, therefore, does not directly measure temporal discounting. Nevertheless, the observed pattern parallels findings from the individual-differences literature: lower metacognitive efficiency is associated with greater impulsivity and reduced pursuit of long-term goals (Rouault, Seow, et al., 2018; Soutschek et al., 2022), and enhancing metacognitive efficiency using frontopolar theta tACS increases preferences for larger, delayed rewards (Soutschek et al., 2021). In our task, higher metacognitive efficiency was associated with better overall performance (cumulative score) and with choices that were less influenced by the magnitude of the per-trial reward offer.

One consideration in interpreting this association is that metacognitive efficiency was estimated from a perceptual discrimination task (Task 2) rather than from the effort task itself. Although metacognition comprises both domain-specific and domain-general components (Fitzgerald et al., 2017; Morales et al., 2018; Pace et al., 2025), reliable cross-domain correlations emerge once cognitive performance is controlled (Lund et al., 2025), and meta cognition has been linked to broad behavioral variations such as impulsivity, attention allocation, effective learning, and the prioritization of task performance (Aguilar-Lleyda et al., 2020; Frömer et al., 2021; Rouault, Seow, et al., 2018; Seow et al., 2021; Yoshida & Saito, 2025). We, therefore, treat the m-ratio as an individual-difference index of a partially domain-general metacognitive component. Because this component is only partly shared across domains, a perceptual estimate would likely attenuate rather than inflate the observed association. Consequently, the present correlation may represent a conservative estimate, and future studies could provide convergent evidence by deriving metacognitive measures directly from the effort task.

The decoding analysis indicated that the neural representation underlying the decision to avoid effort emerged around 260 ms, suggesting that neural processing begins considerably earlier than the actual response (approximately 1.0 s). Additionally, temporal generalization analysis revealed that processing during the early stage (260–400 ms) was time specific and transient, whereas later processing became increasingly generalized over time. This observation suggests that the neural representation of effort-avoidance decisions became more temporally stable over time, a pattern consistent with the maintenance and integration of information before the final response (King & Dehaene, 2014). These findings and interpretations are broadly consistent with models proposing that decision making arises from the continuous accumulation of evidence (Lin et al., 2020; Shenhav et al., 2018; Zhang et al., 2022).

Our RSA and searchlight analyses provide further insights into the temporal dynamics of decision making. By separating the information into reward and difficulty components, we found that reward information was processed earlier than difficulty information. Spatially, reward processing was primarily associated with parietal and occipital electrodes. By contrast, difficulty processing engaged a broader network that included frontal areas while remaining centered on the parietal region. These findings align with behavioral studies, including eye-tracking and choice-trajectory studies using drift diffusion models (DDMs), which suggest that reward is evaluated first, followed by effort or other long-term costs (Sullivan et al., 2015; Westbrook et al., 2020). Similarly, previous EEG research has shown that reward prediction precedes attention-related adjustments (Schevernels et al., 2014). Furthermore, the parietal cortex is widely recognized as a major site of evidence accumulation (Seideman et al., 2022; Zhang et al., 2022).

The present study is significant because it provides direct neural evidence for these temporal dynamics and supports the central role of the parietal region in effort-based decision making. However, recent animal studies suggest that reward, effort, and choice may be processed in a parallel, multistage manner (Härmson et al., 2025). Therefore, future research should integrate noninvasive human neuroimaging approaches with cellular-level findings from animal studies to achieve a more comprehensive understanding of the neural mechanisms underlying decision making.

Because reward and difficulty were signaled by specific visual features, we performed several control analyses to test whether these representations might reflect low-level stimulus processing rather than value- and effort-related computations. A direct model comparison indicated that the early neural geometry was better explained by reward magnitude than by the perceptual distance between the reward-signaling colors, and the decision representations were further dissociable from button-press motor preparation.

The most informative test, however, examined the subject specificity of the representation. Because the cues were physically identical across participants, any purely stimulus-driven signal can contribute only a component shared across individuals, whereas subjective value differs between individuals. We, therefore, examined whether each participant’s neural RDM was better explained by their own subjective-value RDM than by those of the other participants. A key implication of this analysis is that the late representation (from ~600 ms onward) reflects each participant’s individually defined subjective value of the offer rather than its physical attributes. This idiosyncratic structure cannot arise from stimulus processing, such as color, numerosity, or task preparation, and instead points to a value-based representation: the offer is encoded according to how the individual values it. Together with the earlier, stimulus-locked encoding of reward magnitude (140–280 ms), these findings suggest a progression from encoding the offer’s physical attributes to a later, individually weighted valuation. Because behavioral reward sensitivity was low in our task, the subject-specific signal most plausibly reflects each participant’s idiosyncratic weighting of effort costs when valuing the offer. These findings support value-based accounts of effort-based decision making (Klein-Flügge et al., 2016; Kurniawan et al., 2013; Shenhav et al., 2017; Westbrook et al., 2020), in which choices are guided by a subjective, individually calibrated cost–benefit estimate.

Finally, we conducted an exploratory analysis to examine whether the neural representations underlying effort-avoidance decision making differ according to metacognitive efficiency. Our results suggest that reward processing may occur earlier in the low-metacognitive-efficiency group than in the high-metacognitive-efficiency group, although this pattern emerged only in the within-group analyses, and a direct between-group comparison revealed no significant time window. This tentative pattern aligns with the negative correlation between metacognitive efficiency and reward sensitivity observed in our behavioral data and is consistent with previous studies linking lower metacognitive ability to impulsivity (Rouault, Seow, et al., 2018). However, given the exploratory nature of this median-split analysis and the absence of a significant direct group difference, these findings require further validation to establish their statistical robustness.

Although the present study reveals important findings, several limitations should be noted. First, reward and difficulty were signaled by uncounterbalanced visual features (color and dot number); although a series of control analyses argued against a low-level visual explanation (see Section 3.4), this remains a limitation of the experimental design. Second, the generalizability of our task parameters remains an open question. Altering reward types, difficulty levels, or effort domains (cognitive vs. physical) could change the underlying decision-making structure. Future research should examine whether our findings reflect general mechanisms or are specific to the present task design. Third, effort-based choices in our task may share variance with constructs such as fatigue and impulsivity. Although we found no clear evidence of fatigue effects, inferences regarding effort preference should, therefore, be drawn with appropriate caution. Finally, although our behavioral sample met the size suggested by the a priori power analysis, the EEG analyses used a smaller sample (n = 30) and were, therefore, less sensitive to subtle effects, including the exploratory between-group comparison. These findings would benefit from replication in larger samples.

In conclusion, this study provides neural evidence for the temporal dynamics of effort-based decision making, showing that, under the task conditions examined, reward-related information was represented earlier than difficulty-related information. Furthermore, our findings highlight a crucial link between metacognition and decision-making behavior. Higher metacognitive efficiency was associated with reduced sensitivity to the offered reward magnitude and with better overall task performance. Considering that cost–benefit computation and metacognitive abilities are often impaired in neurological and psychiatric disorders, understanding how these functions interact to shape behavior is crucial. To achieve a more comprehensive understanding of the neural mechanisms underlying human decision making, future studies should employ more sophisticated neuroimaging approaches and investigate clinical populations.

## Data Availability

The data are available from the corresponding author (K. Y.) upon reasonable request. Custom scripts for the main analyses are available through the following OSF repository: https://doi.org/10.17605/OSF.IO/7EGV5

## References

[IMAG.a.1332-b1] Aguilar-Lleyda, D., Lemarchand, M., & de Gardelle, V. (2020). Confidence as a priority signal. Psychological Science, 31(9), 1084–1096. 10.1177/0956797620925039 32755439

[IMAG.a.1332-b2] Aridan, N., Malecek, N. J., Poldrack, R. A., & Schonberg, T. (2019). Neural correlates of effort-based valuation with prospective choices. NeuroImage, 185, 446–454. 10.1016/j.neuroimage.2018.10.051 30347281 PMC6289638

[IMAG.a.1332-b3] Bang, D., & Fleming, S. M. (2018). Distinct encoding of decision confidence in human medial prefrontal cortex. Proceedings of the National Academy of Sciences of the United States of America, 115(23), 6082–6087. 10.1073/pnas.1800795115 29784814 PMC6003322

[IMAG.a.1332-b4] Bartra, O., McGuire, J. T., & Kable, J. W. (2013). The valuation system: A coordinate-based meta-analysis of BOLD fMRI experiments examining neural correlates of subjective value. NeuroImage, 76, 412–427. 10.1016/j.neuroimage.2013.02.063 23507394 PMC3756836

[IMAG.a.1332-b5] Basten, U., Biele, G., Heekeren, H. R., & Fiebach, C. J. (2010). How the brain integrates costs and benefits during decision making. Proceedings of the National Academy of Sciences of the United States of America, 107(50), 21767–21772. 10.1073/pnas.0908104107 21118983 PMC3003102

[IMAG.a.1332-b6] Baumeister, R. F., Bratslavsky, E., Muraven, M., & Tice, D. M. (1998). Ego depletion: Is the active self a limited resource? Journal of Personality and Social Psychology, 74(5), 1252–1265. 10.1037/0022-3514.74.5.1252 9599441

[IMAG.a.1332-b7] Blankertz, B., Lemm, S., Treder, M., Haufe, S., & Müller, K.-R. (2011). Single-trial analysis and classification of ERP components—a tutorial. NeuroImage, 56(2), 814–825. 10.1016/j.neuroimage.2010.06.048 20600976

[IMAG.a.1332-b8] Bogdanov, M., & Pizzagalli, D. A. (2025). Shared and distinct features of common effort-based decision-making paradigms and their relation to brain structure and neuropsychiatric conditions: An integrated narrative review. Cognitive, Affective & Behavioral Neuroscience, 26(2), 339–381. 10.3758/s13415-025-01353-6 PMC12671926 41324845

[IMAG.a.1332-b9] Brassard, S. L., Liu, H., Dosanjh, J., MacKillop, J., & Balodis, I. (2024). Neurobiological foundations and clinical relevance of effort-based decision-making. Brain Imaging and Behavior, 18(5), 1–30. 10.1007/s11682-024-00890-x 38819540

[IMAG.a.1332-b10] Chang, C.-C., & Lin, C.-J. (2003). LIBSVM: A library for support vector machines. https://www.cs.cmu.edu/~pakyan/compbio/references/Chang_LIBSVM_2001.pdf

[IMAG.a.1332-b11] David, L., Vassena, E., & Bijleveld, E. (2024). The unpleasantness of thinking: A meta-analytic review of the association between mental effort and negative affect. Psychological Bulletin, 150(9), 1070–1093. 10.1037/bul0000443 39101924

[IMAG.a.1332-b12] Fitzgerald, L. M., Arvaneh, M., & Dockree, P. M. (2017). Domain-specific and domain-general processes underlying metacognitive judgments. Consciousness and Cognition, 49, 264–277. 10.1016/j.concog.2017.01.011 28222381

[IMAG.a.1332-b13] Fleming, S. M. (2017). HMeta-d: Hierarchical Bayesian estimation of metacognitive efficiency from confidence ratings. Neuroscience of Consciousness, 2017(1), nix007. 10.1093/nc/nix007 29877507 PMC5858026

[IMAG.a.1332-b14] Fleming, S. M., Dolan, R. J., & Frith, C. D. (2012). Metacognition: Computation, biology and function. The Royal Society. 10.1098/rstb.2012.0021 PMC3318771 22492746

[IMAG.a.1332-b15] Fleur, D. S., Bredeweg, B., & van den Bos, W. (2021). Metacognition: Ideas and insights from neuro- and educational sciences. NPJ Science of Learning, 6(1), 13. 10.1038/s41539-021-00089-5 34103531 PMC8187395

[IMAG.a.1332-b16] Frömer, R., Nassar, M. R., Bruckner, R., Stürmer, B., Sommer, W., & Yeung, N. (2021). Response-based outcome predictions and confidence regulate feedback processing and learning. eLife, 10, e62825. 10.7554/elife.62825 33929323 PMC8121545

[IMAG.a.1332-b17] Gilbert, S. J., Bird, A., Carpenter, J. M., Fleming, S. M., Sachdeva, C., & Tsai, P.-C. (2020). Optimal use of reminders: Metacognition, effort, and cognitive offloading. Journal of Experimental Psychology. General, 149(3), 501–517. 10.1037/xge0000652 31448938

[IMAG.a.1332-b18] Grootswagers, T., Wardle, S. G., & Carlson, T. A. (2017). Decoding dynamic brain patterns from evoked responses: A tutorial on multivariate pattern analysis applied to time series neuroimaging data. Journal of Cognitive Neuroscience, 29(4), 677–697. 10.1162/jocn_a_01068 27779910

[IMAG.a.1332-b19] Härmson, O., Grennan, I., Perry, B., Toth, R., McNamara, C. G., Denison, T., Cagnan, H., Manohar, S. G., Walton, M. E., & Sharott, A. (2025). Multi-level encoding of reward, effort, and choice across the frontal cortex and basal ganglia during cost-benefit decision-making. Cell Reports, 44(2), 115209. 10.1016/j.celrep.2024.115209 39847484 PMC11860760

[IMAG.a.1332-b20] Hu, X., Luo, L., & Fleming, S. M. (2019). A role for metamemory in cognitive offloading. Cognition, 193(104012), 104012. 10.1016/j.cognition.2019.104012 31271925 PMC6838677

[IMAG.a.1332-b21] Khalighinejad, N., Garrett, N., Priestley, L., Lockwood, P., & Rushworth, M. F. S. (2021). A habenula-insular circuit encodes the willingness to act. Nature Communications, 12(1), 6329. 10.1038/s41467-021-26569-1 PMC8566457 34732720

[IMAG.a.1332-b22] King, J.-R., & Dehaene, S. (2014). Characterizing the dynamics of mental representations: The temporal generalization method. Trends in Cognitive Sciences, 18(4), 203–210. 10.1016/j.tics.2014.01.002 24593982 PMC5635958

[IMAG.a.1332-b23] Klein-Flügge, M. C., Kennerley, S. W., Friston, K., & Bestmann, S. (2016). Neural signatures of value comparison in human cingulate cortex during decisions requiring an effort-reward trade-off. The Journal of Neuroscience: The Official Journal of the Society for Neuroscience, 36(39), 10002–10015. 10.1523/jneurosci.0292-16.2016 27683898 PMC5039251

[IMAG.a.1332-b24] Kurniawan, I. T., Guitart-Masip, M., Dayan, P., & Dolan, R. J. (2013). Effort and valuation in the brain: The effects of anticipation and execution. The Journal of Neuroscience: The Official Journal of the Society for Neuroscience, 33(14), 6160–6169. 10.1523/jneurosci.4777-12.2013 23554497 PMC3639311

[IMAG.a.1332-b25] Kurzban, R., Duckworth, A., Kable, J. W., & Myers, J. (2013). An opportunity cost model of subjective effort and task performance. The Behavioral and Brain Sciences, 36(6), 661–679. 10.1017/s0140525x12003196 24304775 PMC3856320

[IMAG.a.1332-b26] Lin, Z., Nie, C., Zhang, Y., Chen, Y., & Yang, T. (2020). Evidence accumulation for value computation in the prefrontal cortex during decision making. Proceedings of the National Academy of Sciences of the United States of America, 117(48), 30728–30737. 10.1073/pnas.2019077117 33199637 PMC7720192

[IMAG.a.1332-b27] Lockwood, P. L., Cutler, J., Drew, D., Abdurahman, A., Jeyaretna, D. S., Apps, M. A. J., Husain, M., & Manohar, S. G. (2024). Human ventromedial prefrontal cortex is necessary for prosocial motivation. Nature Human Behaviour, 8(7)¸1403–1416. 10.1038/s41562-024-01899-4 PMC11272586 38802539

[IMAG.a.1332-b28] Lockwood, P. L., Wittmann, M. K., Nili, H., Matsumoto-Ryan, M., Abdurahman, A., Cutler, J., Husain, M., & Apps, M. A. J. (2022). Distinct neural representations for prosocial and self-benefiting effort. Current Biology, 32(19), 4172-4185.e7. 10.1016/j.cub.2022.08.010 36029773 PMC9616728

[IMAG.a.1332-b29] Lorenz-Spreen, P., Mønsted, B. M., Hövel, P., & Lehmann, S. (2019). Accelerating dynamics of collective attention. Nature Communications, 10(1), 1759. 10.1038/s41467-019-09311-w PMC6465266 30988286

[IMAG.a.1332-b30] Lund, A. E., Corrêa, C. M. C., Fardo, F., Fleming, S. M., & Allen, M. G. (2025). Domain generality in metacognitive ability: A confirmatory study across visual perception, episodic memory, and semantic memory. Journal of Experimental Psychology. Learning, Memory, and Cognition, 51(10), 1594–1605. 10.1037/xlm0001462 40354297

[IMAG.a.1332-b31] Maniscalco, B., & Lau, H. (2012). A signal detection theoretic approach for estimating metacognitive sensitivity from confidence ratings. Consciousness and Cognition, 21(1), 422–430. 10.1016/j.concog.2011.09.021 22071269

[IMAG.a.1332-b32] Mensen, A., & Khatami, R. (2013). Advanced EEG analysis using threshold-free cluster-enhancement and non-parametric statistics. NeuroImage, 67, 111–118. 10.1016/j.neuroimage.2012.10.027 23123297

[IMAG.a.1332-b33] Mobbs, D., Trimmer, P. C., Blumstein, D. T., & Dayan, P. (2018). Foraging for foundations in decision neuroscience: Insights from ethology. Nature Reviews. Neuroscience, 19(7), 419–427. 10.1038/s41583-018-0010-7 29752468 PMC6786488

[IMAG.a.1332-b34] Morales, J., Lau, H., & Fleming, S. M. (2018). Domain-general and domain-specific patterns of activity supporting metacognition in human prefrontal cortex. The Journal of Neuroscience: The Official Journal of the Society for Neuroscience, 38(14), 3534–3546. 10.1523/jneurosci.2360-17.2018 29519851 PMC5895040

[IMAG.a.1332-b35] Müller, T., Husain, M., & Apps, M. A. J. (2022). Preferences for seeking effort or reward information bias the willingness to work. Scientific Reports, 12(1), 19486. 10.1038/s41598-022-21917-7 36376340 PMC9663561

[IMAG.a.1332-b36] Müller, T., Klein-Flügge, M. C., Manohar, S. G., Husain, M., & Apps, M. A. J. (2021). Neural and computational mechanisms of momentary fatigue and persistence in effort-based choice. Nature Communications, 12(1), 4593. 10.1038/s41467-021-24927-7 PMC8319292 34321478

[IMAG.a.1332-b37] Oosterhof, N. N., Connolly, A. C., & Haxby, J. V. (2016). CoSMoMVPA: Multi-modal multivariate pattern analysis of neuroimaging data in Matlab/GNU Octave. Frontiers in Neuroinformatics, 10, 27. 10.3389/fninf.2016.00027 27499741 PMC4956688

[IMAG.a.1332-b38] Pace, T., Darrant, M., Hermens, D. F., & Andrews, S. C. (2025). Neural oscillations of metacognition: Evidence for domain-specificity and age-related compensation. Cerebral Cortex, 35(10), bhaf285. 10.1093/cercor/bhaf285 41159703 PMC12570022

[IMAG.a.1332-b39] Peirce, J., Gray, J. R., Simpson, S., MacAskill, M., Höchenberger, R., Sogo, H., Kastman, E., & Lindeløv, J. K. (2019). PsychoPy2: Experiments in behavior made easy. Behavior Research Methods, 51(1), 195–203. 10.3758/s13428-018-01193-y 30734206 PMC6420413

[IMAG.a.1332-b40] Qiu, L., Su, J., Ni, Y., Bai, Y., Zhang, X., Li, X., & Wan, X. (2018). The neural system of metacognition accompanying decision-making in the prefrontal cortex. PLoS Biology, 16(4), e2004037. 10.1371/journal.pbio.2004037 29684004 PMC5933819

[IMAG.a.1332-b41] Rahnev, D. (2025). A comprehensive assessment of current methods for measuring metacognition. Nature Communications, 16(1), 701. 10.1038/s41467-025-56117-0 PMC11735976 39814749

[IMAG.a.1332-b42] Rahnev, D., & Fleming, S. M. (2019). How experimental procedures influence estimates of metacognitive ability. Neuroscience of Consciousness, 2019(1), niz009. 10.1093/nc/niz010 31198586 PMC6556214

[IMAG.a.1332-b43] Rouault, M., McWilliams, A., Allen, M. G., & Fleming, S. M. (2018). Human metacognition across domains: Insights from individual differences and neuroimaging. Personality Neuroscience, 1, e17. 10.1017/pen.2018.16 30411087 PMC6217996

[IMAG.a.1332-b44] Rouault, M., Seow, T., Gillan, C. M., & Fleming, S. M. (2018). Psychiatric symptom dimensions are associated with dissociable shifts in metacognition but not task performance. Biological Psychiatry, 84(6), 443–451. 10.1016/j.biopsych.2017.12.017 29458997 PMC6117452

[IMAG.a.1332-b45] Saleh, Y., Jarratt-Barnham, I., Petitet, P., Fernandez-Egea, E., Manohar, S. G., & Husain, M. (2023). Negative symptoms and cognitive impairment are associated with distinct motivational deficits in treatment resistant schizophrenia. Molecular Psychiatry, 28(11), 4831–4841. 10.1038/s41380-023-02232-7 37626135 PMC10914595

[IMAG.a.1332-b46] Schevernels, H., Krebs, R. M., Santens, P., Woldorff, M. G., & Boehler, C. N. (2014). Task preparation processes related to reward prediction precede those related to task-difficulty expectation. NeuroImage, 84, 639–647. 10.1016/j.neuroimage.2013.09.039 24064071 PMC3863725

[IMAG.a.1332-b47] Seideman, J. A., Stanford, T. R., & Salinas, E. (2022). A conflict between spatial selection and evidence accumulation in area LIP. Nature Communications, 13(1), 4463. 10.1038/s41467-022-32209-z PMC9343639 35915096

[IMAG.a.1332-b48] Seow, T. X. F., Jelinek, L., Moritz, S., & Hauser, T. U. (2025). Integrating cognitive neuroscience and clinical perspectives on metacognitive mechanisms in psychopathology. Nature Reviews Psychology, 5(1), 9–28. 10.1038/s44159-025-00503-4

[IMAG.a.1332-b49] Seow, T. X. F., Rouault, M., Gillan, C. M., & Fleming, S. M. (2021). How local and global metacognition shape mental health. Biological Psychiatry, 90(7), 436–446. 10.1016/j.biopsych.2021.05.013 34334187

[IMAG.a.1332-b50] Shenhav, A., Musslick, S., Lieder, F., Kool, W., Griffiths, T. L., Cohen, J. D., & Botvinick, M. M. (2017). Toward a rational and mechanistic account of mental effort. Annual Review of Neuroscience, 40(1), 99–124. 10.1146/annurev-neuro-072116-031526 28375769

[IMAG.a.1332-b51] Shenhav, A., Straccia, M. A., Musslick, S., Cohen, J. D., & Botvinick, M. M. (2018). Dissociable neural mechanisms track evidence accumulation for selection of attention versus action. Nature Communications, 9(1), 2485. 10.1038/s41467-018-04841-1 PMC6021379 29950596

[IMAG.a.1332-b52] Silvestrini, N., Musslick, S., Berry, A. S., & Vassena, E. (2023). An integrative effort: Bridging motivational intensity theory and recent neurocomputational and neuronal models of effort and control allocation. Psychological Review, 130(4), 1081–1103. 10.1037/rev0000372 35679204

[IMAG.a.1332-b53] Soutschek, A., Bulley, A., & Wittekind, C. E. (2022). Metacognitive deficits are associated with lower sensitivity to preference reversals in nicotine dependence. Scientific Reports, 12(1), 19787. 10.1038/s41598-022-24332-0 36396945 PMC9671892

[IMAG.a.1332-b54] Soutschek, A., Moisa, M., Ruff, C. C., & Tobler, P. N. (2021). Frontopolar theta oscillations link metacognition with prospective decision making. Nature Communications, 12(1), 3943. 10.1038/s41467-021-24197-3 PMC8225860 34168135

[IMAG.a.1332-b55] Sullivan, N., Hutcherson, C., Harris, A., & Rangel, A. (2015). Dietary self-control is related to the speed with which attributes of healthfulness and tastiness are processed. Psychological Science, 26(2), 122–134. 10.1177/0956797614559543 25515527 PMC4372728

[IMAG.a.1332-b56] Tadel, F., Bock, E., Niso, G., Mosher, J. C., Cousineau, M., Pantazis, D., Leahy, R. M., & Baillet, S. (2019). MEG/EEG group analysis with brainstorm. Frontiers in Neuroscience, 13, 76. 10.3389/fnins.2019.00076 30804744 PMC6378958

[IMAG.a.1332-b57] Walker, E. Y., Pohl, S., Denison, R. N., Barack, D. L., Lee, J., Block, N., Ma, W. J., & Meyniel, F. (2023). Studying the neural representations of uncertainty. Nature Neuroscience, 26(11), 1857–1867. 10.1038/s41593-023-01444-y 37814025

[IMAG.a.1332-b58] Walther, A., Nili, H., Ejaz, N., Alink, A., Kriegeskorte, N., & Diedrichsen, J. (2016). Reliability of dissimilarity measures for multi-voxel pattern analysis. NeuroImage, 137, 188–200. 10.1016/j.neuroimage.2015.12.012 26707889

[IMAG.a.1332-b59] Westbrook, A., & Braver, T. S. (2015). Cognitive effort: A neuroeconomic approach. Cognitive, Affective & Behavioral Neuroscience, 15(2), 395–415. 10.3758/s13415-015-0334-y PMC4445645 25673005

[IMAG.a.1332-b60] Westbrook, A., van den Bosch, R., Määttä, J. I., Hofmans, L., Papadopetraki, D., Cools, R., & Frank, M. J. (2020). Dopamine promotes cognitive effort by biasing the benefits versus costs of cognitive work. Science (New York, N.Y.), 367(6484), 1362–1366. 10.1126/science.aaz5891 32193325 PMC7430502

[IMAG.a.1332-b61] Yoshida, K., & Saito, R. (2025). The strength of confidence is involved in controlling the intensity of attentional allocation. Scientific Reports, 15(1), 2688. 10.1038/s41598-025-86160-2 39837927 PMC11751452

[IMAG.a.1332-b62] Yoshida, K., Sawamura, D., Ogawa, K., Mototani, T., Ikoma, K., & Sakai, S. (2023). Prospective and retrospective metacognitive abilities and their association with impaired self-awareness in patients with traumatic brain injury. Journal of Cognitive Neuroscience, 35(12), 1960–1971. 10.1162/jocn_a_02064 37788321

[IMAG.a.1332-b63] Zhang, Z., Yin, C., & Yang, T. (2022). Evidence accumulation occurs locally in the parietal cortex. Nature Communications, 13(1), 4426. 10.1038/s41467-022-32210-6 PMC9339004 35907908

